# Supplementary material for: Expression of Cell-Adhesion Molecules in E. coli: A High Throughput Screening to Identify Paracellular Modulators
Source: Int J Mol Sci. 2023 Jun 6;24(12):9784. doi: 10.3390/ijms24129784 (PMC10297866; doi:10.3390/ijms24129784)
Supplement: Supplementary file 1 [file ijms-24-09784-s001.zip › ijms-2428920-supplementary.pdf]

Expression of Cell-Adhesion Molecules in *E. coli*: A High Throughput Screening to Identify Paracellular Modulators

Supplementary Materials

Figure S1. Amino acid sequences and DNA sequences of genes used in this study

pEET28a- OmpW (His tag). Cloned between NcoI and XhoI.

ccatgggaaaaaagttgactgttgcagccctggcggtcactacgctgttatccgggtcggcattcgccc
atgaggccggagagtttttatgcgtgctggaagcgccaccgtccgtccgacagagggcgccggagg
aacgctgggttcgcttggtgggttttccgttacgaataatactcagcttggtttaacatttacctacatggcta
cagacaatatcggagtagagctgttggctgcgacgcctttccgccataaaatcggaactcgcgcaaca
ggtgacatcgccacagttcatcatttaccacctacgctgatggcgcaatggtactttggcgacgcaagttc
taaattccgtccttatgtgggggctggtatcaattacactacgttcttcgataatggattcaatgatcatggg
aaggaagcaggcttgagtgatctttcgctgaaggactcctgggggcggctggacaggttggggtgga
ctacttgatcaatcgcgactggcttgtaaatatgtcagtgtggtacatggacatcgacactactgccaact
acaaattgggtggggcgcagcaacatgactcagtgcgccttgacccctgggtttttatgtttagcgcagg
gtaccgcttcggtgacgtgatcaaa**ctcgag**

```
        10         20         30         40         50         60
MGKKLTVAAL AVTTLLSGSA FAHEAGEFFM RAGSATVRPT EGAGGTLGSL GGFSVTNNTQ

        70         80         90        100        110        120
LGLTFTYMAT DNIGVELLAA TPFRHKIGTR ATGDIATVHH LPPTLMAQWY FGDASSKFRP

       130        140        150        160        170        180
YVGAGINYTT FFDNGFNDHG KEAGLSDLSL KDSWGAAGQV GVDYLINRDW LVNMSVWYMD

       190        200        210        220
IDTTANYKLG GAQQHDSVRL DPWVFMFSAG YRFGDVIKLE
```

Figure S2

pET28a- OmpW-hCLDN1 (His tag). Entire fusion cloned between NcoI and XhoI, an NdeI site was placed in amino acids 219 and 220 for easy substitution with other targets (NdeI toXhoI).

CCATGGGG AAA AAG CTG ACC GTG GCA GCG CTT GCA GTG ACC ACC CTT CTT AGT GGG AGT GCA TTT GCC CAT GAG GCC GGT GAG TTC TTC ATG CGT GCG GGC TCC GCC ACC GTC CGC CCA ACT GAG GGC GCA GGC GGG ACC TTA GGT TCC TTA GGA GGG TTT TCG GTG ACA AAC AAC ACG CAA CTG GGT CTT ACG TTT ACT TAT ATG GCT ACT GAT AAC ATT GGC GTA GAA CTT TTA GCT GCC ACA CCA TTC CGC CAT AAA ATC GGG ACA CGT GCT ACC GGA GAC ATT GCC ACC GTG CAC CAT CTT CCT CCA ACC CTG ATG GCC CAG TGG TAT TTT GGG GAT GCT TCC AGT AAG TTC CGC CCG TAC GTT GGC GCG GGG ATT AAT TAC ACA ACT TTC TTC GAC AAT GGT TTC AAC GAC CAT GGA AAG GAA GCC GGG TTG TCT GAC CTT TCT CTT AAG GAT TCC TGG GGT GCA GCG GGT CAG GTA GGA GTA GAC TAC CTG ATT AAC CGT GAT TGG TTG GTG AAT ATG TCA GTC TGG TAT ATG GAT ATT GAC ACA ACC GCT AAC TAC AAG TTA GGG GGT GCA CAG CAG CAC GAC TCT GTT CGT CTG GAT CCC TGG GTT TTT ATG TTC TCG GCC GGG TAC CGT TTC GGT GGA TCC GGG GGT CAT ATG GCA AAT GCA GGA CTG CAA CTT TTA GGA TTC ATC TTA GCT TTT TTG GGA TGG ATT GGG GCC ATC GTC TCG ACT GCT TTG CCC CAA TGG CGC ATC TAT TCA TAC GCA GGA GAT AAT ATT GTT ACT GCA CAA GCA ATG TAT GAA GGA TTG TGG ATG AGT TGT GTC AGC CAG AGT ACC GGG CAG ATT CAG TGT AAG GTG TTC GAT TCC CTT CTG AAC CTT TCC TCA ACA CTG CAA GCA ACT CGT GCC CTG ATG GTC GTC GGC ATC TTG CTT GGT GTA ATT GCT ATT TTT GTT GCT ACA GTC GGT ATG AAA TGT ATG AAG TGC CTT GAA GAC GAT GAA GTG CAG AAA ATG CGT ATG GCG

GTG ATC GGG GGT GCG ATT TTT CTT CTG GCT GGC TTA GCG ATT CTT GTT GCC

ACC GCC TGG TAC GGC AAC CGC ATC GTA CAG GAG TTC TAC GAC CCT ATG ACT

CCC GTA AAC GCG CGC TAT GAA TTT GGT CAA GCG TTA TTC ACC GGT TGG GCT

GCT GCC TCA TTG TGT TTG TTG GGT GGT GCG TTA CTT TGC TGT TCT TGC CCA

CGC AAA ACA ACC AGC TAT CCT ACG CCC CGC CCT TAC CCC AAA CCT GCT CCC

TCG TCC GGC AAA GAT TAT GTA **CTCGAG**

```
        10         20         30         40         50         60
MGKKLTVAAL AVTTLLSGSA FAHEAGEFFM RAGSATVRPT EGAGGTLGSL GGFSVTNNTQ

        70         80         90        100        110        120
LGLTFTYMAT DNIGVELLAA TPFRHKIGTR ATGDIATVHH LPPTLMAQWY FGDASSKFRP

       130        140        150        160        170        180
YVGAGINYTT FFDNGFNDHG KEAGLSDLSL KDSWGAAGQV GVDYLINRDW LVNMSVWYMD

       190        200        210        220        230        240
IDTTANYKLG GAQQHDSVRL DPWVFMFSAG YRFGGSGGHM ANAGLQLLGF ILAFLGWIGA

       250        260        270        280        290        300
IVSTALPQWR IYSYAGDNIV TAQAMYEGLW MSCVSQSTGQ IQCKVFDSLL NLSSTLQATR

       310        320        330        340        350        360
ALMVVGILLG VIAIFVATVG MKCMKCLEDD EVQKMRMAVI GGAIFLLAGL AILVATAWYG

       370        380        390        400        410        420
NRIVQEFYDP MTPVNARYEF GQALFTGWAA ASLCLLGGAL LCCSCPRKTT SYPTPRPYPK

       430
PAPSSGKDYV LEHHHHHH
```

OmpW in black

hCLDN1= red

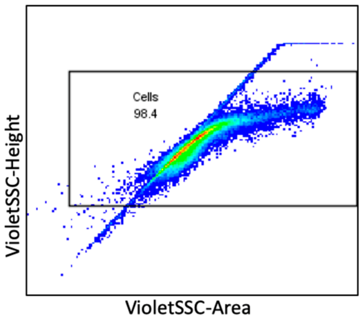

Figure S3. Gating of area and height readings. Data was gated prior to determining the slope of the line to eliminate the upper and lower ends of the data. At least 98% of the readings were included in the gate for analysis.

Figure S4- Chimera DsbB-CLDN5

GCTAGC**CATATG**GAATTCTTACGTTTTCTTAACCAATGTTCTCAGGGTC
GCGGAGCATGGCTTTTGATGGCATTCACGGCGTTAGCCTTGGAATTAAC
AGCCCTGATGTGGCAGGTTACCGCCTTCTTAGATCATAACATTGTGACT
GCTCAGACGACATGGAAAGGTCTTTGGATGTCTTGTGTGGTTCAATCTA
CAGGGCACATGCAATGCAAAGTTTATGACTCCGTTCTTGCGCTGAGCAC
AGAGGTCCAAGCAGCCCGTGCTCTTTTTGGTGTATTGGGTGCGGCATTG
ATTGGAGCAATCGCCCCCAAAACCCCGTTACGCTATGTAGCGATGGTA
ATCTGGTTGTACAGCGCGTTCCGTGGAGTACAACTGACCTACGTGCGTG
AGTTCTATGACCCATCAGTTCCCGTGTCACAGAAGTACGAGTGGCTGTT
GGGCATCTTTATCGCATACTTAATTGTCGCGGTCTTAGTTGTAATTTCGC
AGCCGTTTAAGGCGAAAAAACGTGACCTTTTCGGACGT**CTCGAG**GGTG
GTGATTACAAGGATGACGACGATAAGTAGTCTAGA

Figure S5

Proteins in this study and their Accession numbers. Proteins were used full length unless specified.

Protein	Accession number	URL
Human CLDN1	O95832	https://www.uniprot.org/uniprot/O95832
Human CLDN2	P57739	https://www.uniprot.org/uniprot/P57739
Human CLDN3	O15551	https://www.uniprot.org/uniprot/O15551
Human CLDN4	O14493	https://www.uniprot.org/uniprot/O14493
Human CLDN5	O00501	https://www.uniprot.org/uniprot/O00501
Human CLDN6	P56747	https://www.uniprot.org/uniprot/P56747
Human CLDN7	O95471	https://www.uniprot.org/uniprot/O95471
Human CLDN8	P56748	https://www.uniprot.org/uniprot/P56748
Human CLDN9	O95484	https://www.uniprot.org/uniprot/O95484
Human CLDN10	P78369	https://www.uniprot.org/uniprot/P78369
Human Tricellulin (TRCL)	Q8N4S9 (Met186-His368)	https://www.uniprot.org/uniprot/Q8N4S9
Human Stargazin (STRGZN)	P62955 (Met1-Arg203)	https://www.uniprot.org/uniprot/P62955

Figure S6

iCLASP identified compounds from the ChemBridge DIVERSet library, furthered selected in A549 lung cancer cells. In Green are compounds that increased proliferation of A549, while in Red are the ones that decreased it. For each compound, the first 3 letters are our lab's identifiers. The seven following digits are identifiers in the ChemBridge website: https://www.hit2lead.com/search.asp

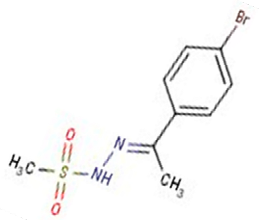

G01:5478603

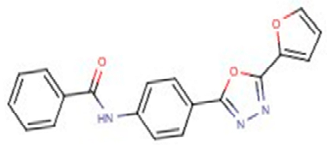

G04:6502939

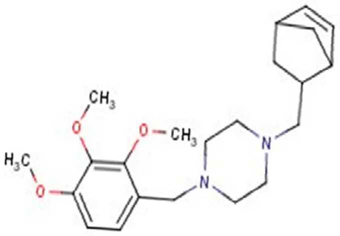

H03:5990019

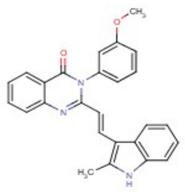

A04: 6000179

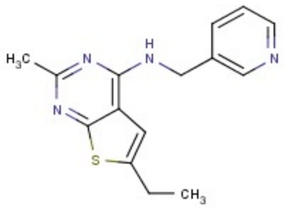

A10:9017768

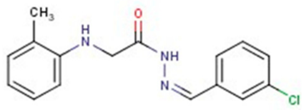

C02:5534282

Figure S7
Drug discovery workflow using iCLASP.

Round 1

- Fresh transformation of BL21 DE3 cells with the plasmid for OmpW-CLDN2 are performed two days before. Colonies are selected from the plate and grown to OD_{600}=1, IPTG is then added to a final concentration of 1 mM. Growth continues overnight (18 h) at room temperature with shaking. Colonies were selected from plates up to 3 weeks after transformation.
- Cells are then transferred to the 96-well plates. The composition of the plates is 180 µL of PBS, 20 µL of compounds, and 50 µL of cells. Plates are prepared in quadruplicates. Each 96-well plate corresponds to ~ 1 h of Flow Cytometry under our conditions and with our equipment and settings.
- The 50,000-compound library was combined in 10-coumpounds per well, without particular order (to a final concentration of10 µM). Thus, each well contained 10 unique compounds, representing the challenge to cells overexpressing OmpW-CLDN2. The Experimental Slope is determined for each challenge after combining the quadruplicate results from that day. Each plate preserves the first and last column for untreated cells overexpressing OmpW-CLDN2 (a total of 16 wells). Experimental controls are compared to the unchallenged control. Decreased slopes represent HYPER-permeability. Increased slopes represent HYPO-permeability.

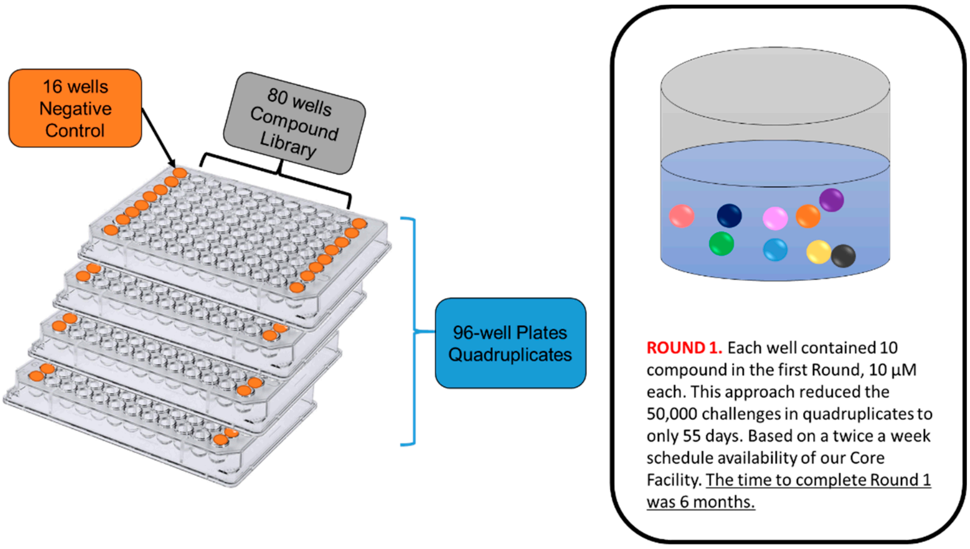

The graphs below represent the highest and lowest increase over the control slope (unchallenged cells) selected for each day where the experiment was performed in quadruplicates as explained above.

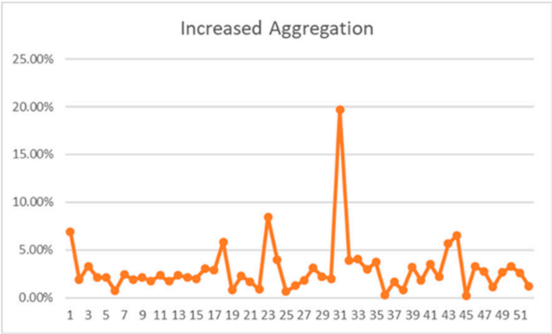

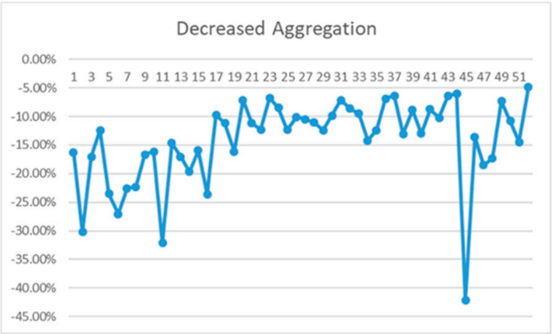

Round 2

- Wells representing 10 unique compounds were identified as causing Hyper or hypopermeability (Round 1). The best challenges, altering permeability higher or lower are selected. Each challenge consisted of 10 compounds. These compounds are now used as challengers in a single-compound fashion. We selected the best 4 challenges that lead to increased slope and the best 4 leading to decrease in slope. Corresponding to 40 compounds in each category.
- From this second round we expect to identify the compounds that correspond to the changes in the slope, and isolate them from the others

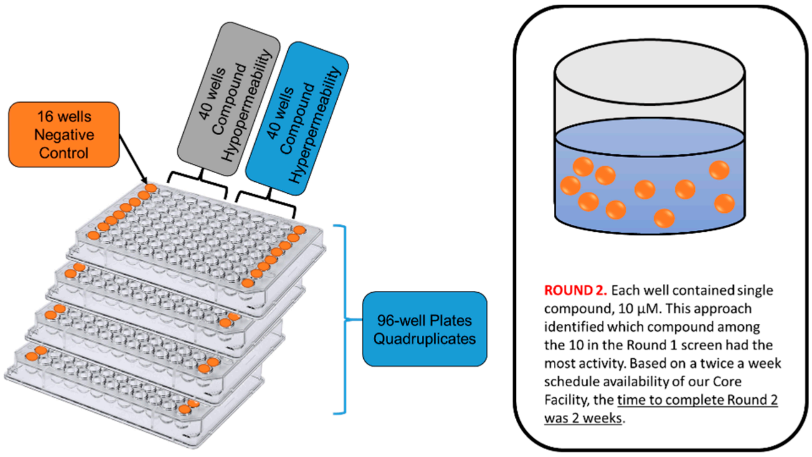

Round 3

- From Round 2 we determined a series of 10 compounds in each category that correspond to the best single-compound challengers to cell-cell interactions leading to the greatest changes compared to the unchallenged cells.
- In Round 3 we prepared dilutions of each compound to challenge cell-cell interactions: 10, 5, 2, 1, 0.8, 0.4, 0.2, 0.1 µM.
- Below we present a graph of one of these compounds (G04:6502939) that demonstrated a dose-dependence.

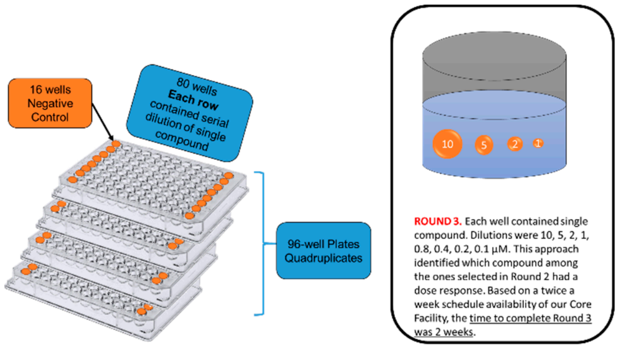

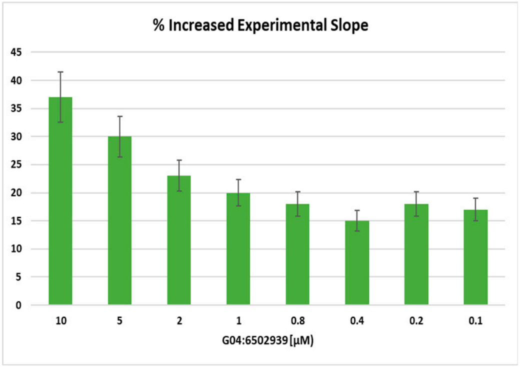

***** Selected compounds were then tested in lung cancer cell line A549 at 10 µM for maximum effect.
